# Intra-individual comparison of long-term outcomes between combined and indirect bypass surgery in adult moyamoya disease

**DOI:** 10.1007/s00701-024-06391-6

**Published:** 2025-01-09

**Authors:** Yuwhan Chung, Jeong Eun Kim, Hyun-Seung Kang, Tae Young Kim, Jin Chul Paeng, Won-Sang Cho, Sung Ho Lee, Eun Jin Ha, Kangmin Kim

**Affiliations:** 1 Department of Neurosurgery, Seoul National University Hospital, Seoul National University College of Medicine, Seoul, Republic of Korea; 2 Department of Nuclear Medicine, Seoul National University Hospital, Seoul National University College of Medicine, Seoul, Republic of Korea; 3 Department of Critical Care Medicine, Seoul National University Hospital, Seoul National University College of Medicine, Seoul, Republic of Korea

**Keywords:** Combined bypass surgery, Indirect bypass surgery, Moyamoya disease, Intra-individual comparison

## Abstract

**Purpose:**

Bypass surgery is regarded as the standard treatment option for symptomatic and hemodynamically unstable moyamoya disease (MMD). However, there is ongoing debate about the most effective type of bypass surgery. We aimed to analyze the long-term outcomes of combined and indirect bypasses for MMD patients through intra-individual comparisons.

**Methods:**

Of the 896 patients who underwent 1084 bypass surgeries between 2007 and 2021, 24 patients with MMD who underwent combined bypass on one side and indirect bypass on the other side were ultimately enrolled in this study. Clinical, angiographic and hemodynamic outcomes were retrospectively evaluated.

**Results:**

Three asymptomatic strokes (12.5%) occurred within 30 postoperative days in each group. Postoperative strokes after 30 days occurred in 3 patients (12.5%) with 3 hemorrhagic events and 1 cerebral infarction, only in indirect bypass, while no stroke occurred in hemispheres treated with combined bypass. The revascularization area relative to supratentorial area was significantly greater in combined bypass than in indirect bypass, both in short-term and long-term periods (64.9% versus 43.9% in short-term and 75.7% versus 54.9% in long-term; *P* < .001, respectively). Hemodynamic outcomes showed significantly greater increases in acetazolamide-challenged cerebral blood flow (CBF_acz_) during short-term follow-up (*P* = .04) and in both basal CBF (CBF_bas_) and CBF_acz_ during long-term follow-up (*P* = .014 and *P* = .009, respectively) in combined bypass than in indirect bypass.

**Conclusion:**

Combined bypass may be a more effective treatment option for MMD based on its higher revascularization area and favorable hemodynamic results compared to indirect bypass in the same patient.

## Introduction

Moyamoya disease (MMD) is a chronic cerebrovascular disease involving the progressive occlusion of the terminal portion of the internal carotid artery and the development of moyamoya vessels [23]. Although revascularization surgery is accepted as the standard treatment for symptomatic and hemodynamically unstable adult MMD patients, ongoing debate persists regarding the optimal types of revascularization surgeries, in contrast to those for pediatric MMD patients [1–4, 9, 19, 21, 24]. Despite meta-analyses and various retrospective studies indicating the superiority of combined bypass over indirect bypass in reducing late strokes and achieving favorable outcomes, limitations exist due to heterogeneity in surgical techniques and selection biases arising from differences among patients [2, 10, 11, 20, 22]. We thus aimed to evaluate long-term outcomes by comparing bilateral MMD patients who underwent combined and indirect bypasses on each hemisphere, using a homogeneous protocol at a single institute.

## Methods

### Patient selection

Bilateral MMD patients who underwent combined and indirect bypasses on each hemisphere respectively between January 2007 and December 2022 were retrospectively reviewed. The inclusion criteria in this study were as follows: (1) underwent combined and indirect bypasses on each hemisphere respectively, aged ≥ 18 years; (2) diagnosed with MMD according to the guidelines of the Research Committee on Moyamoya Disease, established by the Japanese Ministry of Health and Welfare [1, 6, 7]; (3) recurrent symptoms such as transient ischemic attack, cerebral infarction and intracranial hemorrhage including intracerebral hemorrhage (ICH) and intraventricular hemorrhage (IVH); (4) significant decreases in basal perfusion and reservoir capacity on brain single photon emission computed tomography (SPECT); (5) functionally independent state with a Karnofsky Performance Scale (KPS) ≥ 70.

**Fig. 1:**
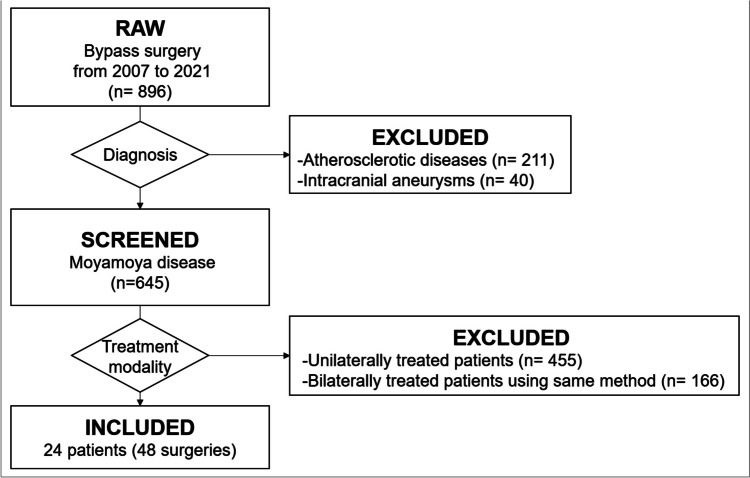
Flow chart showing the inclusion criteria

A total of 1086 bypass surgeries were performed on 896 patients, among which 211 patients with intracranial atherosclerotic steno-occlusive diseases and 40 patients with intracranial aneurysms were excluded. Out of 645 MMD patients, 455 patients who were treated unilaterally and 166 patients who were treated bilaterally using the same surgical method were excluded. Finally, 24 patients who underwent 48 bypasses (24 combined bypasses and 24 indirect bypasses on each hemisphere) were enrolled in this study (Fig. 1). The study was approved by the institutional review board (2402-010-1506), exempting the need for informed consent from the patients.

The general postoperative follow-up schedule was as follows: (1) hospitalization for digital subtraction angiography (DSA), SPECT, and perfusion magnetic resonance imaging (MRI) at 6 months (short-term period) and 5 years (long-term period) after surgery; (2) alternating annual perfusion MRI and SPECT up to 5 years after surgery, and then every two years in the outpatient setting.

### Surgical procedures

Combined bypass, including superficial temporal artery (STA)–middle cerebral artery (MCA) anastomosis and encephalodurogaleosynangiosis (EDGS), is generally performed for adult MMD patients, as described in previous literature [3]. Indirect bypass, including encephaloduroarteriogaleosynangiosis, was indicated in the following cases: (1) patients with profuse transdural collaterals via the STA that cannot be sacrificed; (2) absence of a proper recipient; (3) poor STA flow.

In combined bypass, direct bypass was performed using the parietal branch of the STA as a donor artery and the cortical branch of the MCA as the recipient. The STA and distal MCA were anastomosed in an end-to-side fashion using prolene 10 − 0 sutures around Chater’s point. After the anastomosis, EDGS was performed as an indirect bypass. The incised dura mater was cut into two to three pieces and folded into the anterior subdural space and a galeal flap was used to cover the exposed brain.

The process of indirect bypass surgery did not significantly differ from that of direct bypass surgery. The key distinction lay in preserving the parietal branch of the STA without cutting and approximately 5 mm of the galeal margin around the STA was maintained. This preserved margin was then sutured to both the incised dura margin and the galeal flap to cover the exposed brain.

All surgical procedures were performed under general anesthesia with mild hypothermia (34 ℃). The mean arterial blood pressure was maintained within a range of ± 10 mmHg from the preoperative baseline during the operation. An antiepileptic drug was administered and maintained for one week perioperatively.

### Clinical evaluations

The baseline characteristics are summarized in Table 1. Combined and indirect bypasses were performed in 48 hemispheres in 24 adult MMD patients at our institution. In 16 patients, the combined bypass was performed before the indirect bypass. The mean interval between the combined and indirect bypasses in a patient was 22.9 ± 34.7 months (range, 0.4–126.5 months). For 17 patients, the interval between the two surgeries was less than one year. There were no significant differences in initial presentation and Suzuki grade between the hemispheres that underwent combined and indirect bypasses (*P* = .808 and *P* = .764, respectively).

**Table 1:** Basal characteristics^* ^

Variables	Value		*P* value
No. of patients	24		
Sex			
Male	4 (16.7)		
Female	20 (83.3)		
Age at time of first surgery, y	35.4 ± 12.6 (18–61)		
Comorbidity			
Hypertension	9 (37.5)		
Diabetes mellitus	0 (0)		
Hyperlipidemia	4 (16.7)		
Current smoker	3 (12.5)		
Time interval between surgeries, mo	22.9 ± 34.7 (0.4–126.5)		
	Combined bypass	Indirect bypass†	
No. of hemispheres	24	24	
Initial presentations			0.808
Ischemia			
Transient ischemic attack	16 (66.7)	15 (62.5)	
Infarction	6 (25.0)	8 (33.3)	
Hemorrhage	2 (8.3)	1 (4.2)	
Preoperative Suzuki grade			0.764
2	4 (16.7)	2 (8.3)	
3	5 (20.8)	5 (20.8)	
4	12 (50.0)	12 (50.0)	
5	3 (12.5)	5 (20.8)	

^*^Data are number of patients (%) for categorical variables and mean ± SD(range) for continuous variables

The patients’ clinical status was assessed using the KPS at admission, 1 month after surgery, and last follow-up. The mean durations of clinical follow-up were 105.3 ± 50.5 months (range, 30–201 months) and 119.4 ± 50.9 months (range, 31–200 months) for combined and indirect bypass, respectively (*P* = .439). Postoperative stroke events were examined within the postoperative 30 days and thereafter, respectively. Additionally, postoperative complications other than stroke were also evaluated. MRI with perfusion and time-of-flight imaging was performed to evaluate the status of patients at the scheduled time points and when neurological symptoms were present. The mean durations of radiological follow-up were 93.0 ± 44.7 months (range, 30–177 months) and 107.2 ± 47.3 months (range, 29–183 months) in combined and indirect bypass, respectively (*P* = .392).

Cerebral hyperperfusion syndrome (CHS) was diagnosed in patients with transient neurologic symptoms after surgery, a hyperperfusion area on SPECT or arterial spin-labeling MRI, and no acute infarction or hemorrhage observed on CT and MRI.

### Angiographic evaluation

DSA was generally performed preoperatively (within 1 month) and postoperatively at 6 months and 5 years. Follow-up DSA was performed to evaluate the patency of STA-MCA anastomosis, revascularization of bypass surgery, and disease progression. The Suzuki grade of MMD was evaluated according to the methods of Suzuki and Kodama [23].

The revascularization area (RA) and supratentorial area were quantitatively measured using MAROSIS PACS system (INFINITT, Seoul, Korea) according to a previously reported method [2, 3]. The relative RA was determined using the following formula: relative RA (%) = RA/supratentorial area × 100. Each area was measured three times and the mean value of these measurements was used to determine the relative RA.

### Hemodynamic evaluation

Hemodynamic evaluation was performed using basal and acetazolamide (Diamox^®^)-challenged SPECT with 99mTc-hexamethylpropyleneamine oxime, which was conducted within 2 months before surgery, and in the short-term (5–12 months) and long-term (30–88 months) periods after surgery. Semiquantitative analysis of the SPECT results was conducted in a manner consistent with a previous report [15]. Images were spatially normalized using statistical parametric mapping (SPM2, University College London, London) implemented in MATLAB (version R2019b, MathWorks Inc., Natick, MA). The mean voxel count of the cerebellum was used as a control cerebral blood flow (CBF) of 50. The mean CBF of the MCA territory in each SPECT image was evaluated using proportional scaling. The cerebrovascular reserve (CVR) was calculated with the basal CBF (CBF_bas_) and acetazolamide-challenged CBF (CBF_acz_) using the following formula: (CBF_acz_ – CBF_bas_)/CBF_bas_ × 100. CBF_acz_ refers to the value acquired during SPECT imaging with acetazolamide administration, while CBF_bas_ indicates the value from a basal SPECT. The increment ratios were calculated using the following formula: (post – pre)/pre × 100 of CBF_acz_, CBF_bas_, and CVR in each follow-up period and each bypass.

### Statistical analysis

Continuous variables are presented as the means ± standard deviations (range) and assessed using an independent t-test or Wilcoxon Rank Sum test (Mann–Whitney U test), as appropriate. For categorical variables, frequencies and percentages were presented, and depending on the expected frequency distribution, either a chi-square test or Fisher’s exact test was performed. A Linear Mixed Model, which accounts for correlation structures between hemodynamic values repeatedly measured at three time points, was conducted. *P* values < 0.05 indicated statistical significance, with a two-sided 95% confidence interval. Statistical analysis was performed using SAS statistical software (SAS system for Windows, version 9.4; SAS Institute Inc., Cary, NC) and SPSS statistics 29.0 (IBM SPSS Inc., Chicago, IL).

## Results

### Clinical outcomes

**Fig. 2:**
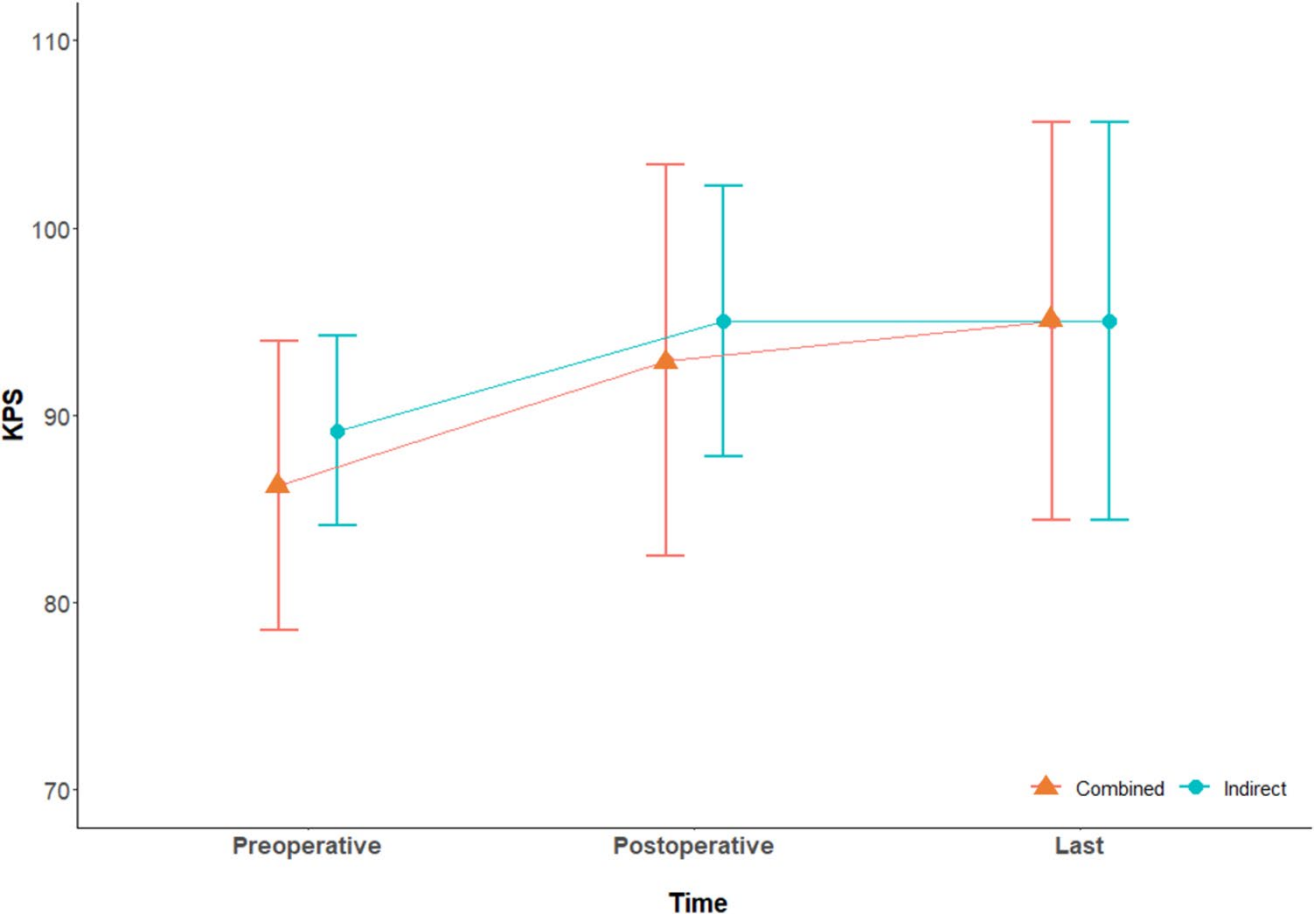
Comparison of clinical status at each period between combined and indirect bypass with the Karnofsky Performance Scale

Clinical states at each period are displayed in Fig. 2. The mean preoperative KPS was 86.3 ± 7.7 (range, 70–90) and 89.2 ± 5.0 (range, 70–100) for combined and indirect bypasses, respectively (*P* = .139). The mean one-month postoperative KPS was 92.9 ± 10.4 (range, 70–100) and 95.0 ± 7.2 (range, 70–100), respectively (*P* = .742). The mean KPS at the last follow-up was 95.0 ± 10.6 (range, 70–100) for the same patients who underwent both bypasses.

**Fig. 3:**
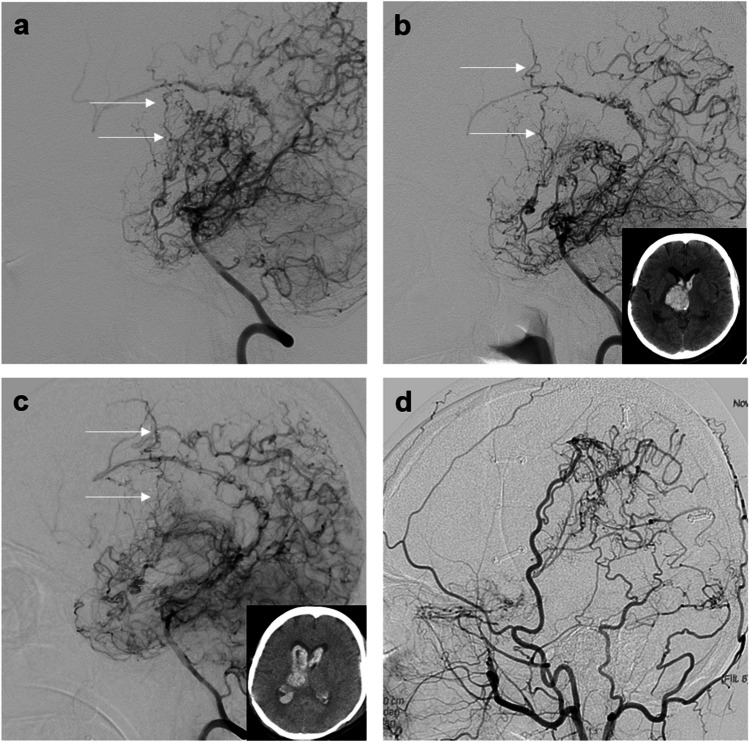

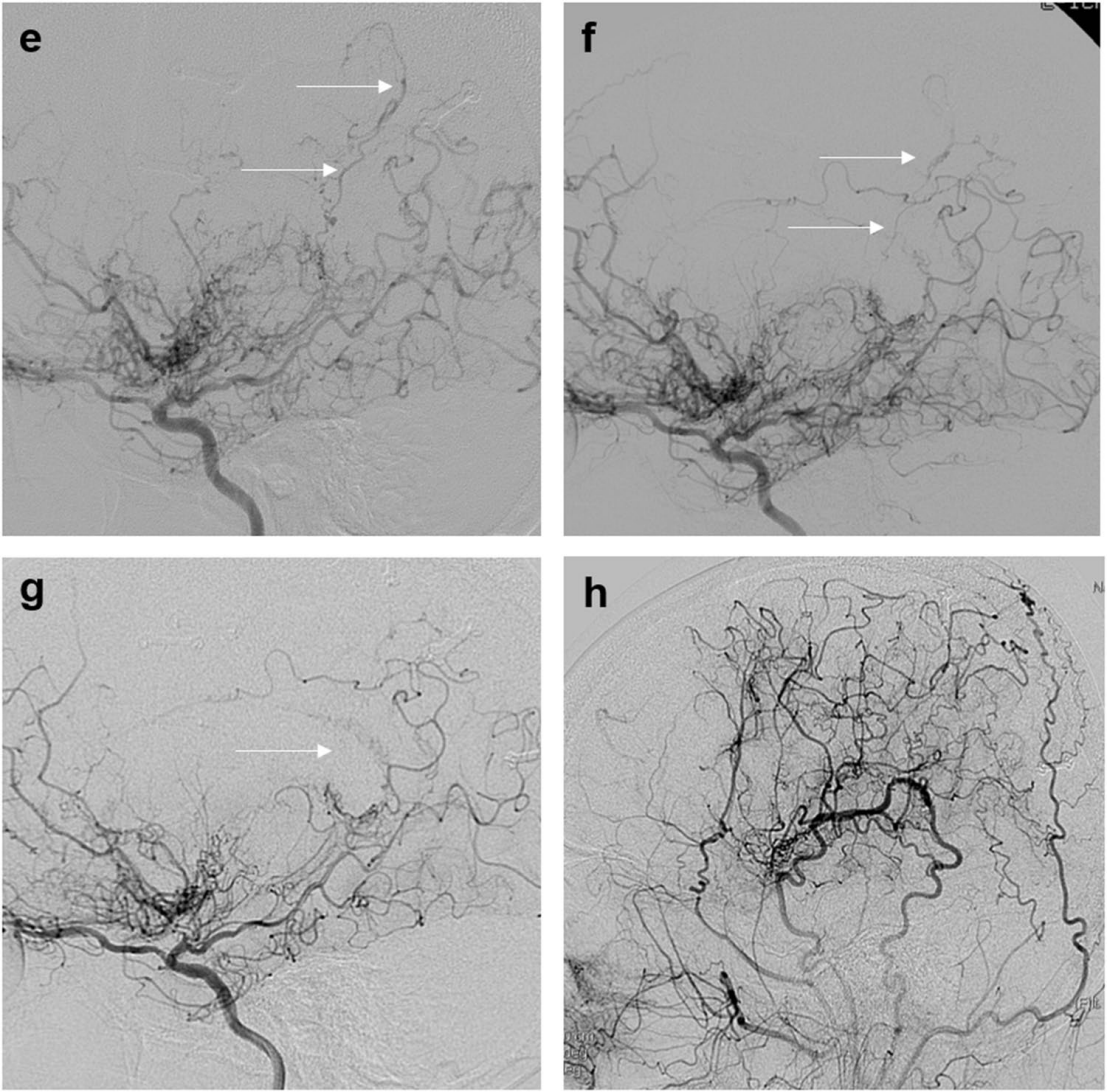
A representative case of periventricular anastomosis in combined and indirect bypass. Lateral view of digital subtraction angiography (DSA) of the vertebral artery in the hemisphere of the indirect bypass demonstrating (**a**), thalamic anastomosis (white arrow), preoperatively. Two consecutive instances of intracerebral hemorrhage with intraventricular hemorrhage confirmed by brain computed tomography with more prominent thalamic anastomosis (white arrow) in (**b**), postoperative 2-year and (**c**), 3-year follow-up DSA. **d**. Revascularization of indirect bypass in postoperative 3-year right external carotid artery DSA. Lateral view of DSA of the left internal carotid artery revealed choroidal anastomosis (white arrow) in the hemisphere of combined bypass (**e**), preoperatively and restoration of the choroidal anastomosis (white arrow) at postoperative (**f**). 6-months and (**g**). 5-year follow-up. **h**. Revascularization of combined bypass on postoperative 5-year follow-up DSA

The clinical outcomes are shown in Table 2. Postoperative stroke within 30 days occurred in 3 patients (12.5%) each in combined and indirect bypasses, all of whom had asymptomatic cerebral infarction (*P* = 1.000). Postoperative stroke after 30 days occurred in 2 patients (8.3%) with 3 hemorrhagic events and in 1 patient (4.2%) with cerebral infarction, which occurred only in indirect bypass (*P* = .234). In patients with cerebral infarction, recovery occurred without sequelae; however, in two patients with hemorrhage, sequelae corresponding to KPS 70 and 80 due to hemorrhage were observed at the last follow-up. Multiple stroke events occurred in only one patient, with two consecutive ICH with IVH observed solely in the hemisphere that underwent indirect bypass (Fig. 3).

**Table 2:** Clinical outcomes^*^

	Combined bypass	Indirect bypass	*P* value
Postoperative complication^†^			0.523
Subdural hemorrhage	0 (0)	2 (8.3)	
Cerebral hyperperfusion syndrome	7 (29.2)	5 (20.8)	
Postoperative stroke within 30 days			1.000
Cerebral infarction	3 (12.5)	3 (12.5)	
Hemorrhage	0 (0)	0 (0)	
Postoperative stroke after 30 days			0.234
Cerebral infarction	0 (0)	1 (4.2)	
Hemorrhage	0 (0)	2 (8.3)	
Follow-up duration, mo			
Clinical results	105.3$\:\pm\:$50.5 (30–201)	119.4$\:\pm\:$50.9 (31–200)	0.439
Radiological results	93.0$\:\pm\:$44.7 (30–177)	107.2$\:\pm\:$47.3 (29–183)	0.392

^*^Data are number of patients (%) for categorical variables and mean ± SD (range) for continuous variables

^†^All the patients with complications were transiently symptomatic and recovered well without permanent sequelae

### Angiographic outcomes

**Fig. 4:**
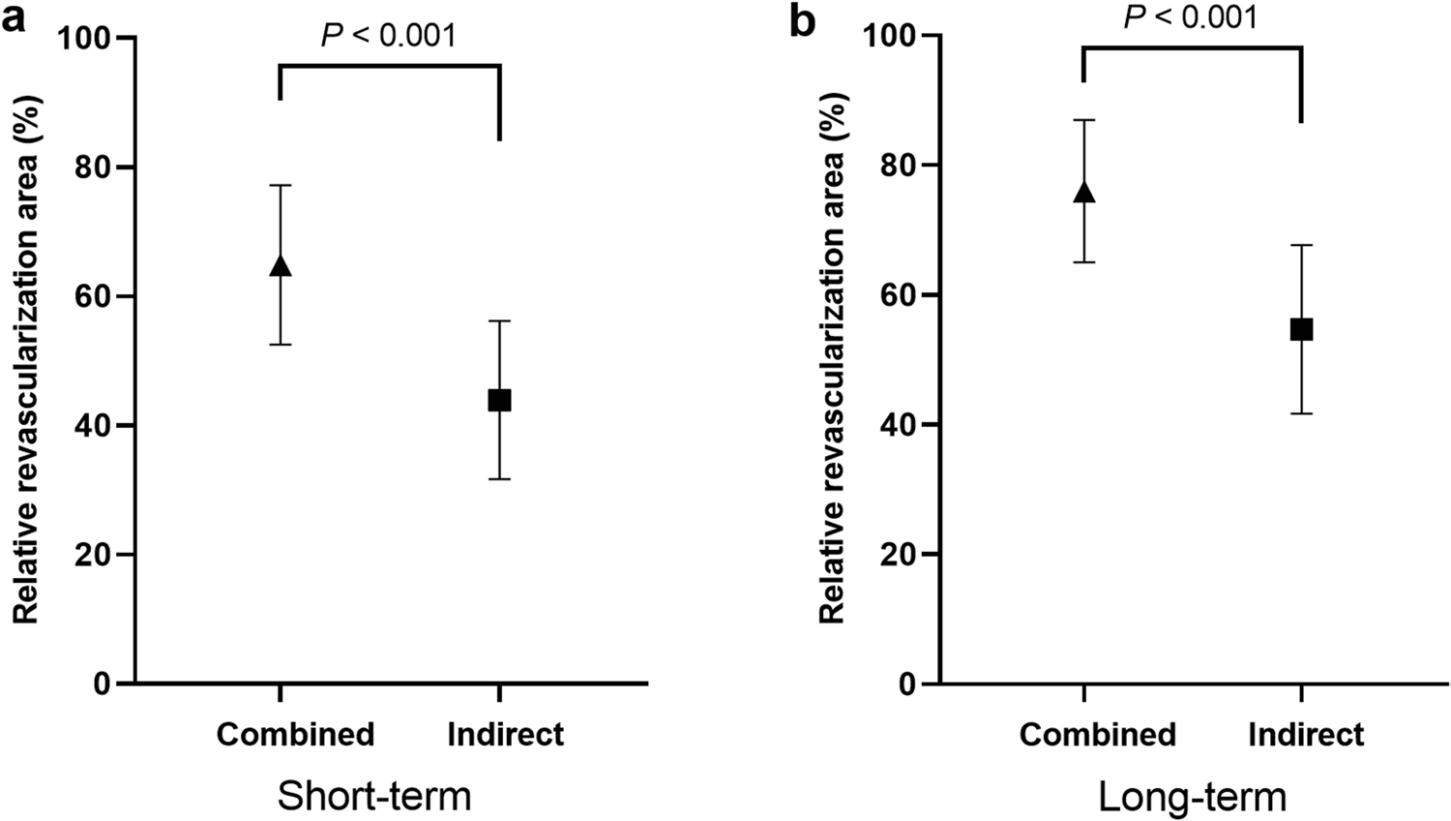
Comparison of relative revascularization areas in the (**a**), short-term and (**b**), long-term follow-up periods between combined and indirect bypass surgery

The angiographic outcomes are presented in Fig. 4. Short-term follow-up angiographies were available for all 24 patients in both combined and indirect bypasses with mean follow-up durations of 6.5 ± 1.0 months (range, 5–9 months) and 6.75 ± 1.6 months (range, 5–12 months), respectively (*P* = 1.000). Long-term follow-up angiographies were available for 21 (87.5%) and 22 (91.7%) patients with a mean follow-up duration of 66.2 ± 7.4 months (range, 59–88 months) and 65.0 ± 9.7 months (range, 56–91 months) in the combined and indirect bypasses, respectively (*P* = .065). In both the short-term and long-term follow-up periods, the relative RA of combined bypass was significantly greater than that of indirect bypass (*P* < .001 for each). The relative RA showed a significant increase by the long-term follow-up compared to the short-term follow-up in both combined and indirect bypasses (*P* = .003 and *P* = .006, respectively).

### Hemodynamic outcomes

**Fig. 5:**
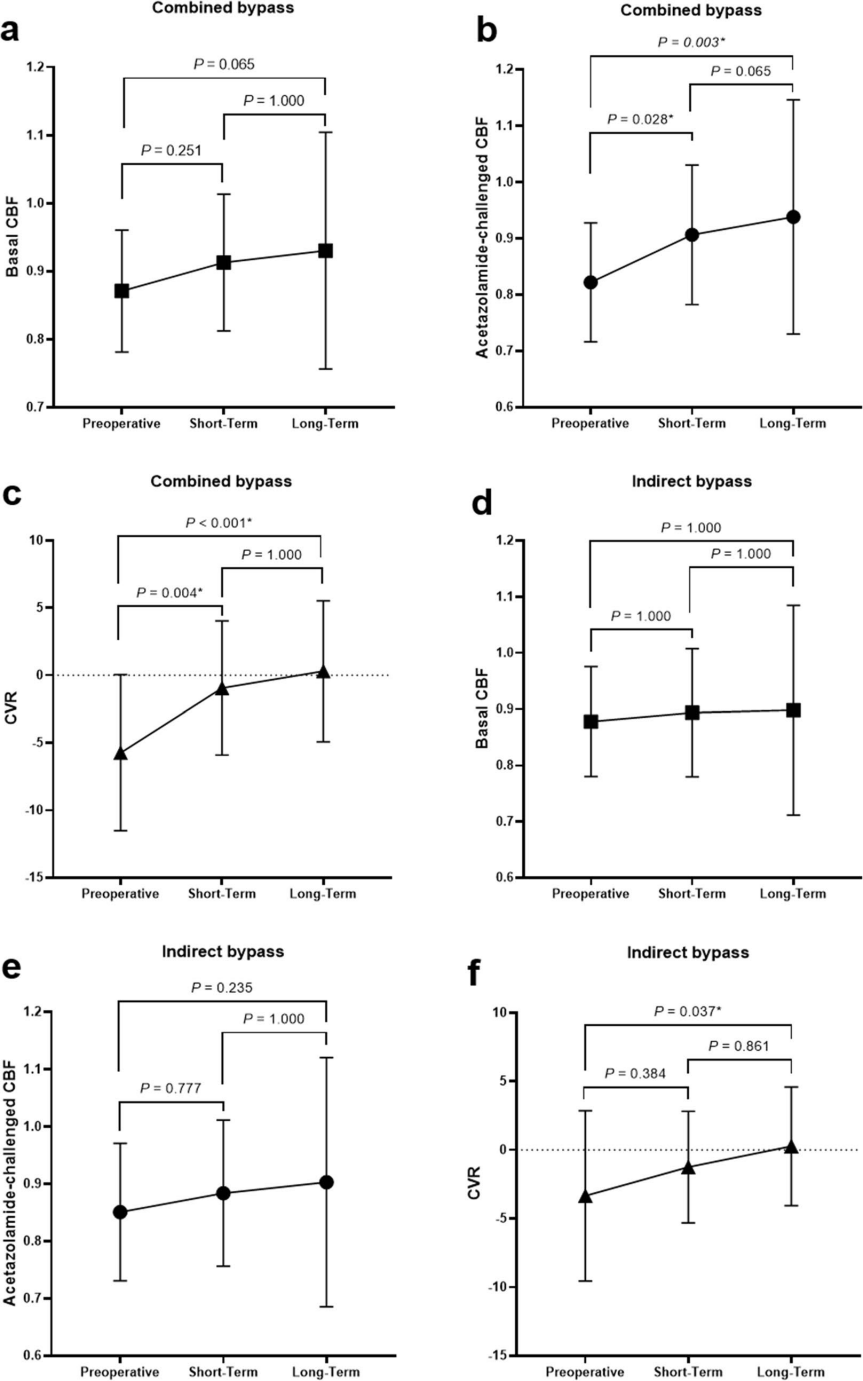
Comparison of the hemodynamic results at each period. **a**, Basal cerebral blood flow (CBF), (**b**), acetazolamide-challenged CBF, and (**c**), cerebrovascular reserve (CVR) in combined bypass. (**d**), basal CBF, (**e**), acetazolamide challenge CBF, and (**f**), CVR in indirect bypass. *P < .05 indicates statistical significance

Semiquantitative analyses of brain SPECT are summarized in Fig. 5. Hemodynamic follow-up with brain SPECT was available for 24 (100%) patients, except for long-term follow-up in combined bypass, for which 23 (95.8%) patients were available. The mean short-term follow-up durations in combined and indirect bypasses were 6.7 ± 1.1 months (range, 5–9 months) and 6.8 ± 1.6 months (range, 5–12 months), respectively (*P* = .836), and 62.3 ± 12.4 months (range, 30–88 months) and 58.6 ± 11.1 months (range, 60–76 months) in the long-term follow-up, respectively (*P* = .054).

The preoperative CBF_bas_, CBF_acz_, and CVR showed no significant differences between the combined and indirect bypasses (*P* = .529, *P* = .177, and *P* = .174, respectively). In the combined bypass, CBF_acz_ significantly improved during both short-term and long-term follow-ups compared with the preoperative period (*P* = .028 and *P* = .003, respectively; Fig. 5b), and CVR also increased significantly (*P* = .004 and *P* < .001, respectively; Fig. 5c). In the indirect bypass, only the CVR between the preoperative and long-term follow-up periods significantly increased (*P* = .037, Fig. 5f). CBF_bas_ did not significantly change between the two periods in either groups (Fig. 5a and d).

The results of the increment ratios are summarized in Table 3. Analyses of short-term increment ratios revealed significant differences in CBF_acz_ between combined and indirect bypasses (*P* = .040). Additionally, in terms of the long-term increment ratios, combined bypass significantly improved both CBF_bas_ and CBF_acz_ compared to indirect bypass (*P* = .014 and *P* = .009, respectively).

**Table 3:** Hemodynamic outcomes^*^

	Combined bypass	Indirect bypass	*P* value
Short-term increment ratio^†^, %			
Basal CBF	5.04 ± 7.59 (−17.92–15.97)	1.88 ± 8.68 (−29.13–14.52)	0.146
Acetazolamide-challenged CBF	10.88 ± 13.01 (−14.73–39.91)	4.16 ± 8.66 (−17.27–20.43)	**0.040**
Cerebrovascular reserve	−239.49 ± 455.22 (−2115.06–83.16)	394.4 ± 1833.12 (−494.64–8884.1)	0.078
Long-term increment ratio^‡^, %			
Basal CBF	6.70 ± 16.95 (−30.74–65.33)	2.01 ± 16.79 (−33.05 − 68.92)	**0.014**
Acetazolamide-challenged CBF	14.50 ± 24.61 (−30.79–99.50)	6.21 ± 21.54 (−23.33–98.20)	**0.009**
Cerebrovascular reserve	−1329.63 ± 5545.93 (−26659.77–344.11)	793.55 ± 4192.04 (−618.45–19978.60)	0.843

^*^Data are mean ± SD (range) for increment ratios

^†^Short-term increment ratio = (Short-term - Preoperative)/Preoperative * 100

^‡^Long-term increment ratio = (Long-term - Preoperative)/Preoperative * 100

Boldface type indicates statistical significance

## Discussion

In this study, 48 hemispheres in 24 patients were treated with combined and indirect bypasses for each hemisphere of the same patient. Postoperative strokes after 30 days occurred in 3 patients (12.5%) including cerebral infarction in 1 patient (4.2%) and ICH with IVH in 2 patients (8.3%), all exclusively in the indirect bypass. Angiographic revascularization increased progressively in both groups up to long-term follow-up, with significantly greater values observed in the combined bypass. In contrast to indirect bypass, both CBF_acz_ and CVR showed statistically significant increases from short-term periods in the combined bypass. Regarding the increment ratio, combined bypass demonstrated superior results compared to indirect bypass. To the best of our knowledge, this study is the first to demonstrate direct and intuitive evidence comparing outcomes between combined and indirect bypasses by eliminating inter-patient bias through intra-individual comparisons.

Although revascularization surgery is acknowledged as the standard treatment for adult MMD patients, there remains controversy regarding the optimal surgical modality [3, 14, 16, 24]. A recent meta-analysis showed that combined bypass demonstrated statistically superior results compared to indirect bypass in late stroke (> 30 days postoperatively) and favorable outcomes [20]. Jeon et al. demonstrated that the incidence of stroke events for combined or direct bypass was 7.7%, while for indirect bypass, it was 16.5%, suggesting that combined or direct bypass showed a significantly lower risk of stroke events [10]. In contrast, a large multicenter study recently reported that there is no significant difference in stroke prevention between direct bypass and indirect bypass [5]. Our intra-individual study showed that late stroke events occurred in 0% of combined bypass and 12.5% of indirect bypass. Given these considerations, it is critical to conduct new large prospective studies with an established protocol to address the limitations of previous studies.

Various methodologies are being proposed to identify which type of surgical modality is the most effective. In our study, we analyzed the results using relative RA and semiquantitative analysis of SPECT. Bang et al. demonstrated a significantly greater relative RA with combined and direct bypasses than with indirect bypass and reported that these values were correlated with the SPECT results [2]. They demonstrated that the mean relative RA at postoperative 6-month angiography was 32.4% in indirect bypass, while it ranged from 57.4 to 70.8% in combined and direct bypass. In our study’s short-term follow-up, indirect bypass had a mean relative RA of 42.3%, while combined bypass showed 64.9%, consistent with trends reported previously. Additionally, it has been reported that the wider the revascularization area posterior to the central sulcus is, the better the reduction in choroidal anastomosis [8]. Based on these previous studies, the results of this study may carry sufficient significance in the correlation between relative RA and hemodynamic results, as well as clinical outcomes.

The Japan Adult Moyamoya trial showed the efficacy of combined and direct bypasses for preventing recurrent hemorrhagic stroke [18]. Kaplan–Meier survival analysis showed a significant difference in the rate of rebleeding (2.7%/y in the surgical group versus 7.6%/y in the nonsurgical group). Additionally, Kuroda et al. showed that markedly developed choroidal anastomosis was associated with the risk for hemorrhagic stroke even in asymptomatic MMD [13]. The importance of periventricular anastomosis and STA-MCA anastomosis was further emphasized by the fact that periventricular anastomosis could be restored after combined and direct bypass [17]. Although one study suggested that indirect bypass leads to the regression of lenticulostriatal anastomosis, it was limited by the small sample size [12]. In our study, there was a patient who experienced recurrent hemorrhage after surgery, with two consecutive instances of ICH with IVH occurring at the second and third years postoperatively in the hemisphere that underwent indirect bypass. The cause of the hemorrhage was identified as a markedly developed thalamic anastomosis, which remained prominent on DSA even at 7.5 years after indirect bypass. On the other hand, in the same patient, choroidal anastomosis was present in the contralateral hemisphere, and after undergoing combined bypass surgery, restoration was confirmed on DSA performed at 6 months postoperatively, with no subsequent stroke events observed. Further studies with larger sample sizes are needed to compare the efficacy of combined and indirect bypass on the regression of periventricular anastomosis.

This study had several limitations. First, it may have included possibilities of inherent selection bias due to its retrospective nature and small sample size. Nevertheless, this study is considered significant because long-term follow-up was conducted with consistent surgical procedures at a single tertiary institution with high experience in bypass surgery. Second, variations in basal characteristics may exist within the same patient due to differences in the timing of bypass surgery and the states of MMD between hemispheres. However, in 17 patients, the interval between combined and indirect bypasses was less than one year. Furthermore, there were no statistically significant differences in the preoperative Suzuki grade and hemodynamic results between both hemispheres. Third, RA and supratentorial area estimations were based on 2-dimensional angiographic images, which do not account for the brain surface’s curvature. Therefore, more precise methodologies should be developed. Further prospective studies are needed to clarify the optimal surgical modality for MMD.

## Conclusion

The findings of this intra-individual comparison study suggest that combined bypass achieves more satisfactory outcomes than indirect bypass because of its greater revascularization area and favorable hemodynamic result. This study strengthens the power of the meta-analysis by reducing heterogeneity. Prospective studies with larger sample sizes are needed to provide further evidence for the best treatment modality.

## Data Availability

All data generated or analyzed during this study are included in this article. Further inquiries can be directed to the corresponding author.

## Abbreviations

CBF: cerebral blood flow
CBF_acz_: acetazolamide-challenged CBF
CBF_bas_: basal CBF
CHS: cerebral hyperperfusion syndrome
CVR: cerebrovascular reserve
DSA: digital subtraction angiography
EDGS: encephalodurogaleosynangiosis
ICH: intracerebral hemorrhage
IVH: intraventricular hemorrhage
KPS: Karnofsky Performance Scale
MCA: middle cerebral artery
MMD: moyamoya disease
MRI: magnetic resonance imaging
RA: revascularization area
SPECT: single-photon emission computed tomography
STA: superficial temporal artery
